# Supplementary material for: Fully printed zero-static power MoS2 switch coded reconfigurable graphene metasurface for RF/microwave electromagnetic wave manipulation and control
Source: Nat Commun. 2024 Dec 4;15:10591. doi: 10.1038/s41467-024-54900-z (PMC11618370; doi:10.1038/s41467-024-54900-z)
Supplement: Supplementary file 1 — Supplementary Information [file 41467_2024_54900_MOESM1_ESM.pdf]

Supplementary Information

Fully printed non-volatile MoS_2 switch coded reconfigurable graphene metasurface for RF/microwave electromagnetic wave manipulation and control.

Xiaoyu Xiao*[1], Zixing Peng*[2], Zirui Zhang[1], Xinyao Zhou[1], Xuzhao Liu[3], Yang Liu[3], Jingjing Wang[2], Haiyu Li[1], Kostya S Novoselov[4,5,6], Cinzia Casiraghi[2], Zhirun Hu **[1,5]

1.Department of Electrical and Electronics, University of Manchester, M13 9PL, UK

2.Department of Chemistry, University of Manchester M13 9PL, UK.

3.Department of Materials, University of Manchester M13 9PL, UK.

4.Department of Physics and Astronomy, University of Manchester, M13 9PL Manchester, United Kingdom

5.National Graphene Institute, University of Manchester, M13 9PL, Manchester, United Kingdom

6.Centre for Advanced 2D materials, National University of Singapore,117546, Singapore

**These authors contributed equally: Xiaoyu Xiao, Zixing Peng*

***Address correspondence to: Zhirun Hu*

Content

S1. Inkjet-printed zero-static power RF/microwave MoS_2 Switches

S1.1 Ag/MoS_2/Ag switch cross-section overview

S1.2 State-of-the-art on printed inorganic resistive switches

S1.3 De-embedding process

S2. Zero-static power MoS_2 switching mechanism.

S3. Proof-of-concept demonstration of fully printed zero-static power MoS_2 switch coded RF/microwave reconfigurable graphene metasurface.

S1. Inkjet-printed zero-static power RF/microwave MoS_2 memristive switches

S1.1 $Ag/MoS_2/Ag$ memristive switch cross-section overview

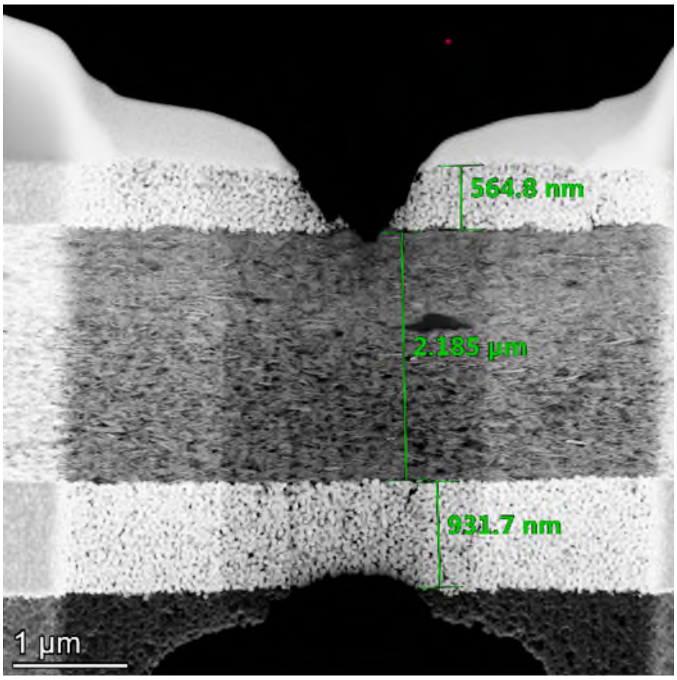

Fig. S1 HAADF-STEM overview image of the zero-static power $Ag/MoS_2/Ag$ memristive switch (20 times on/off cycles). The bright dotted layers are Ag electrodes, and the middle grey layer is MoS_2 dielectric layer.

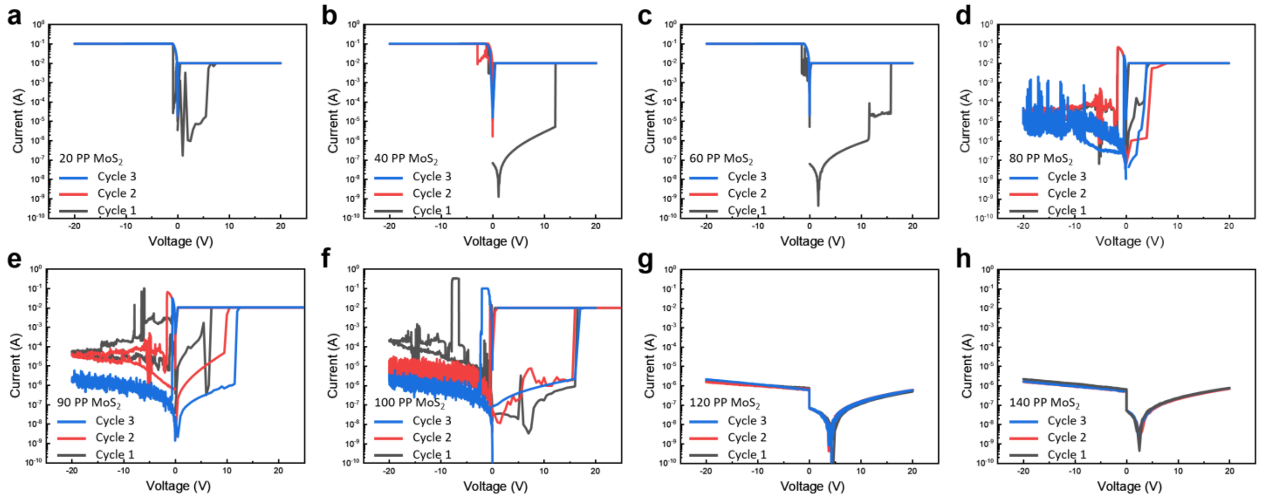

Fig. S2 Memristive switching characteristics of $Ag/MoS_2/Ag$ switches, tested over 3 cycles, made with 2 mg/mL MoS_2 ink and printed with different printing passes (PPs): (a) 20 PPs; (b) 40 PPs; (c) 60 PPs; (d) 80 PPs; (e) 90 PPs; (f) 100 PPs; (g) 120 PPs; (h) 140 PPs.

S1.2 State-of-the-art of printed inorganic memristive switches

Table SI. State-of-the-art memristive switches fabricated by solution-based inorganic materials using printing methods.

Structure	Printing technique	LRS resistance	Set/Reset voltage	Cycle endurance	Testing duration	Lifetime	Ref
$Ag/MoS_2/Ag$	Inkjet printing	10^1 Ω	Set 1.75 V; Reset -0.7 V	300 cycles	10^7 s	10 years	Our work
Ag/ZnO/Ag	Electrohydrodynamic printing, spin coating	10^6 Ω	Set 2 V; Reset -2 V	N/A	N/A	N/A	1
Ag/ZnO/Cu	Electrohydrodynamic Printing	10^3 Ω	Set 1.25 V; Reset -1.25 V	500 cycles	N/A	N/A	2
$Ag/TiO_2/Cu$	Electrohydrodynamic Printing	10^2 Ω	Set 0.7 V; Reset -0.7 V	N/A	N/A	N/A	3
$Ag/ZnSnO_3/$ Ag	Screen Printing, Electrohydrodynamic Atomization	10^7 Ω	Set 2 V; Reset -2 V	100 cycles	10^5 s	N/A	4
$Au/Cu\text{-}SiO_2$ NWs/Cu	Aerosol-Jet Printing	10^4 Ω	Set 3 V; Reset -3 V	10^4 cycles	10^6 s	10 years	5
Ag/a-TiO_2/Ag	Inkjet Printing	10^7 Ω	Set 10 V; Reset -10 V	10^3 cycles	100 s	N/A	6
Ag/ZnO/Ag	Electrohydrodynamic Printing	10^2 Ω	Set 3.75 V; Reset -3.75 V	10^3 cycles	N/A	N/A	7
$Ag/ZrO_2/Ag$	Electrospray Deposition, Electrohydrodynamic Printing	10^2 Ω	Set 3.8 V; Reset -2.6 V	N/A	N/A	N/A	8
Ag/h-BN/Ag	Inkjet Printing	10^5 Ω	Set 2 V; Reset -1 V	10^5 cycles	10^4 s	N/A	9
$Ag/MoS_2/Ag$	Aerosol-Jet Printing	10^1 Ω	Set 0.18 V - 0.30 V, Reset -0.1 V	100 cycles	10^5 s	N/A	10
$Ag/WSe_2/Ag$	Aerosol-Jet Printing, Pneumatic atomizer	10^5 Ω	Set 0.7 V; Reset -0.25 V	N/A	N/A	N/A	11
Ag/TiO_2/Carbon	Screen Printing, Inkjet Printing	10^3 Ω	Set 1 V; Reset -3 V	100 cycles	N/A	N/A	12
$Ag/ZrO_2/Ag$	Electrohydrodynamic Printing	10^5 Ω	Set 3 V; Reset -3 V	100 cycles	N/A	N/A	13
Ag/Cr-N-doped TiO_2/Ag	Reverse offset printing, EHD Printing, EHDA Printing	10^4 Ω	Set 1 V; Reset -1 V	500 cycles	5000 s	N/A	14
Ag/MoS_2/Gr	Inkjet printing	10^4 Ω	Set 2 V; Reset -0.24 V	100 cycles	10^5 s	10 years	15

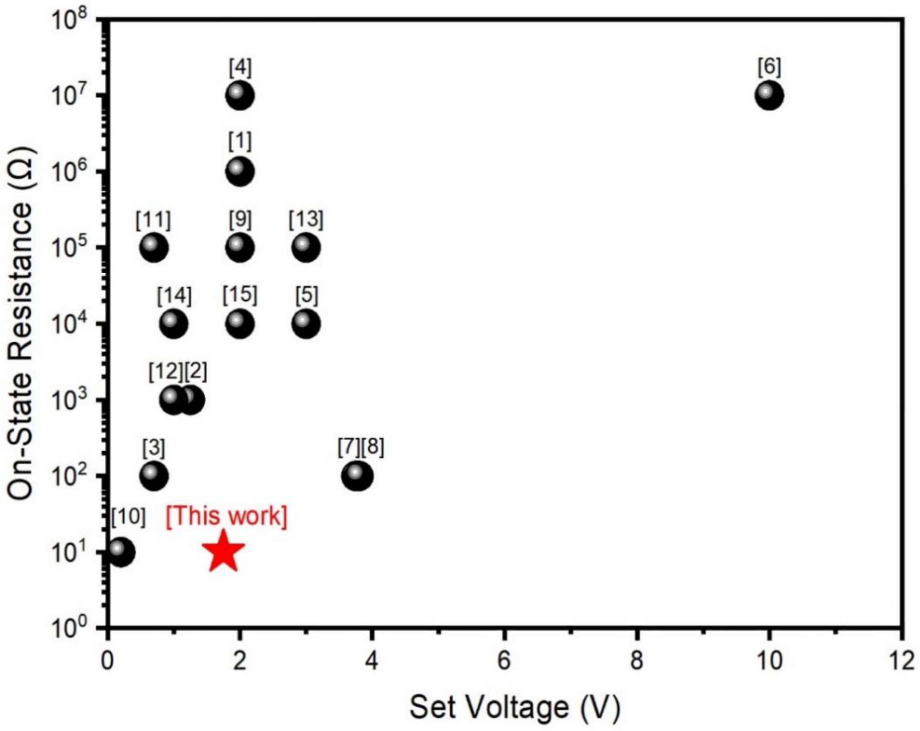

Fig. S3 Benchmarking of the on-state resistance of the Ag/MoS_2/Ag switch compared to state-of-the-art printed inorganic memristive switches. The use of non-volatile switches can greatly increase the energy efficiency for reconfigurable systems, especially when a system has a large number of switches, as such switches do not dissipate static power to maintain states. Furthermore, for RF/microwave antenna and circuit applications, it is highly desirable to have as low ON-state resistance as possible so to ensure low insertion loss and low energy consumption. This figure shows that this printed device has achieved the lowest ON-state resistance in the printed inorganic memristive switches so far.

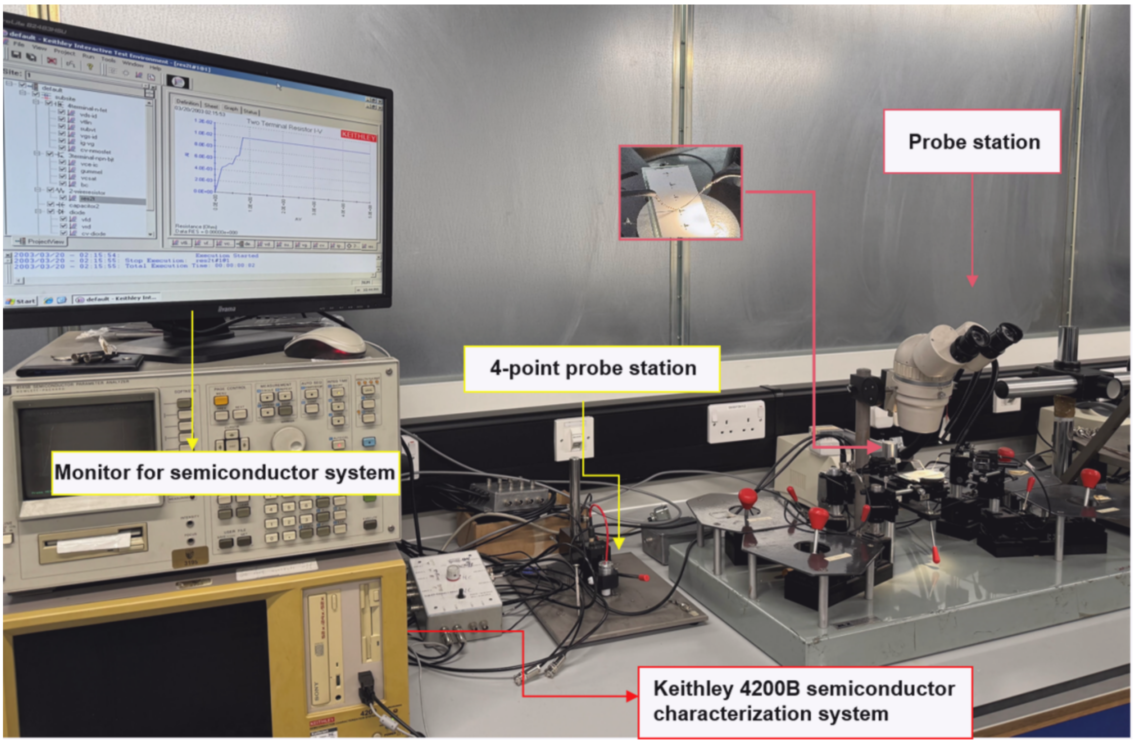

Fig. S4 Device measurement set up.

FieldFox Vector Network Analyzer N9918A was used for measuring RF properties of Ag/MoS_2 /Ag memristive switchs, shown in Fig. S5. For the metasurface, the bias was provided through Agilent 33210A Function Waveform Generator through the bias line on the metasurface. The sheet resistance of the graphene layer was measured using 4-point probe station (Jandel, RM3000).

S1.3 De-embedding process

To obtain precise element values in the equivalent circuit model and eliminate extrinsic effects caused by test cables, de-embedding process that utilizes test samples was conducted to obtain the intrinsic S-parameters of non-volatile MoS_2 RF/microwave switches by removing the probe-pad and interconnect resistance and other parasitic effects by the subtract and SMAs. We've tested the fixture with non-volatile MoS_2 switch and the stand-alone microstrip line, which can be seen in Fig. 2(a) and Fig. S5, respectively. First the S-parameters matrix obtained by the test fixture was converted into T-parameters matrix. The microstrip line T matrix was extracted. The microstrip line was deliberately designed to be symmetrical. In this way, as shown from Equations S (1) to (4), the effect of microstrip line can be de-embedded.

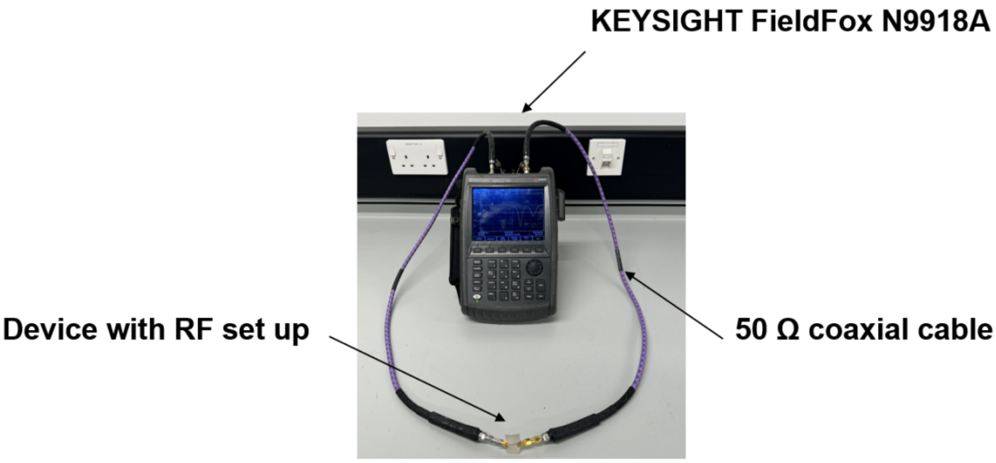

Fig. S5 RF measurement set up.

$$\begin{bmatrix} T_{11} & T_{12} \\ T_{21} & T_{22} \end{bmatrix} = \left(\frac{1}{S_{21}}\right) \begin{bmatrix} S_{12}S_{21} - S_{11}S_{22} & S_{11} \\ -S_{22} & 1 \end{bmatrix} \quad \text{S(1)}$$

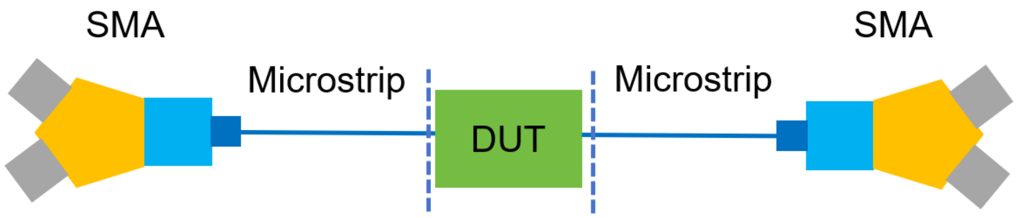

Fig. S6 Diagram of non-volatile MoS_2 switch with microstrip line and SMA.

$$[T_{Measurement}] = [T_{Microstrip\ line\ left}] * [T_{Dut}] * [T_{Microstrip\ line\ right}] \quad \text{S(2)}$$

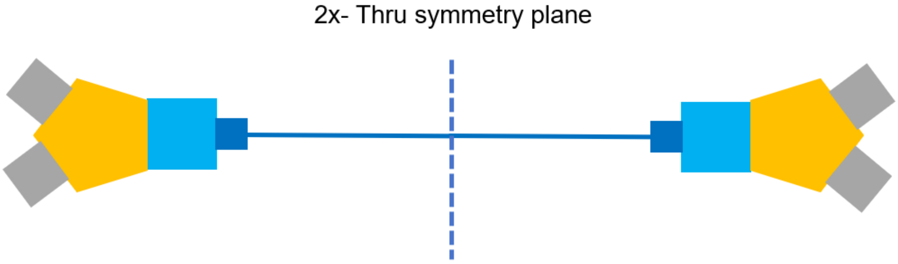

Fig. S7 Diagram of only microstrip line and SMA

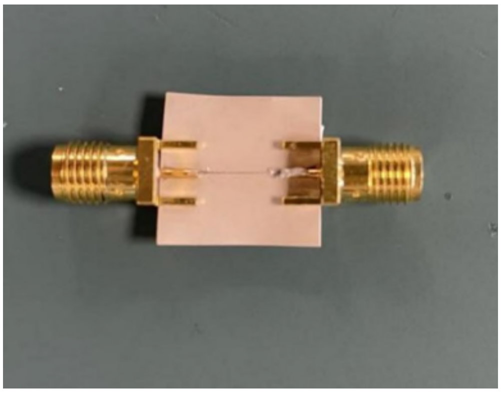

Fig. S8 Photo of fabricated microstrip line and SMA

$$[T_{Measurement\ Thro}] = [T_{Microstrip\ lineleft}] * [T_{Microstrip\ line\ right}] \quad S(3)$$

$$[T_{Measurement-Dut}] = [T_{Microstrip\ line\ left}]^{-1} * [T_{Measurement}] * [T_{Microstrip\ line\ right}]^{-1} \quad S(4)$$

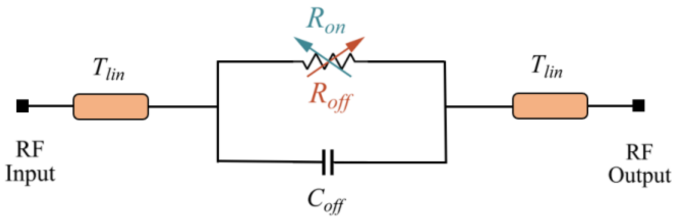

Fig. S9 Model of the MoS_2 switch showing switching element along with various parasitic circuit components.

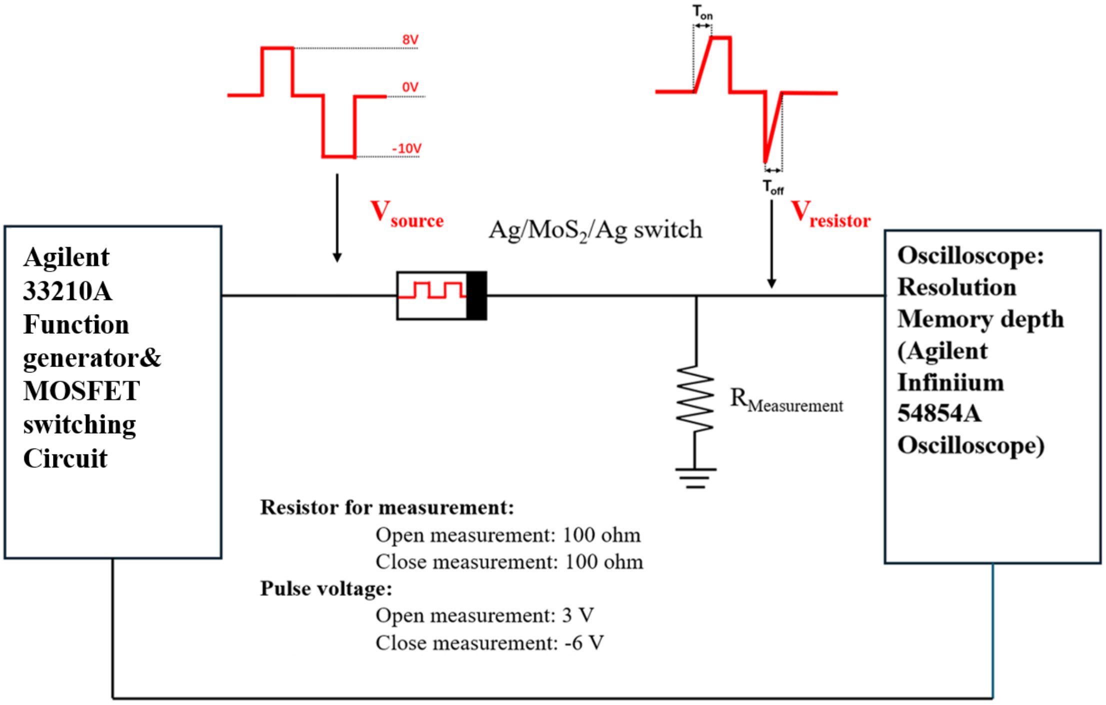

Fig. S10 Switching speed measurement setup.

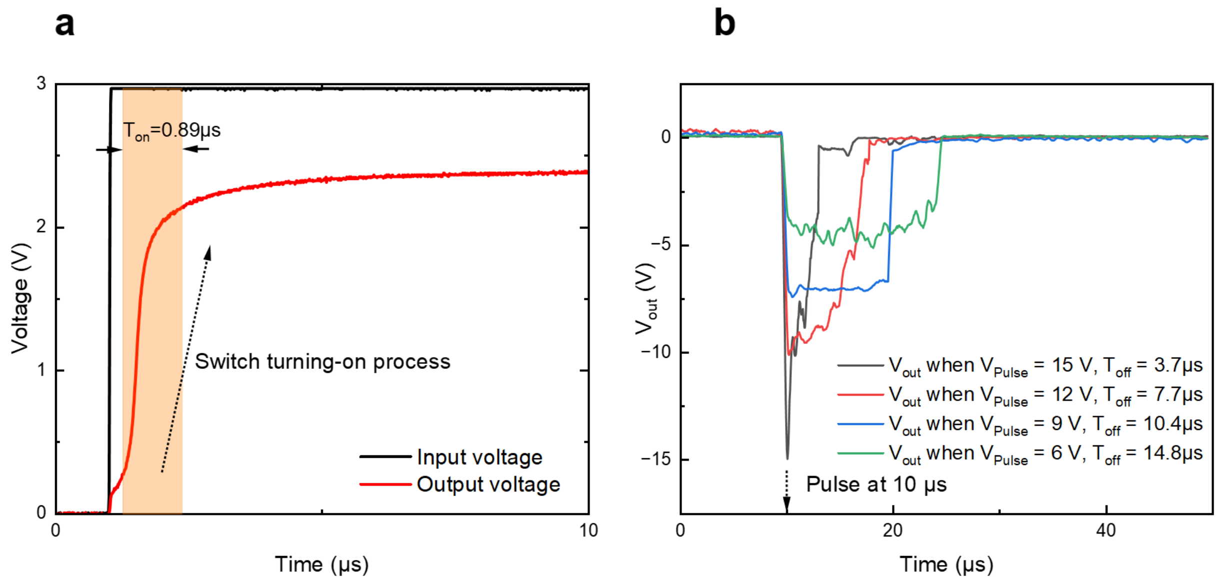

Fig. S11 (a) Applied and responding pulse voltages of an Ag/MoS_2/Ag device (switch ON) and (b) switch OFF with different pulse voltages.

The schematic diagram of switching speed measurement setup is depicted in Fig. S10. The measurement setup involves monitoring the voltage across the resistor connected in series and the source voltage from the Agilent 33210A Function Generator and the IRF520 MOSFET switching circuit. For both turn-on and turn-off assessments, the series resistors are set at 100 Ω.

Fig. S11 (a) displays the turn-on waveform. The 100 Ω series resistor serves to limit the current and protect the system. The Ag/MoS2/Ag device activates with a rise time of approximately 0.89 μs. Fig. S11 (b) depicts the output voltage changes of the switch over time following a turn-off pulse initiated at 10 μs. Each voltage trace exhibits a sharp negative spike at a specific moment, after which it remains steady for a period before instantly returning to zero, indicating the switch's closing moment. The turn-off time varies from 3.7 to 14.8 μs. Notably, the turn-off time depends on the magnitude of the applied turn-off pulse, lengthening as the pulse voltage decreases. Increasing the turn-off pulse voltage can decrease the turn-off time.

S2. Zero-static power MoS_2 memrisitve switching mechanism.

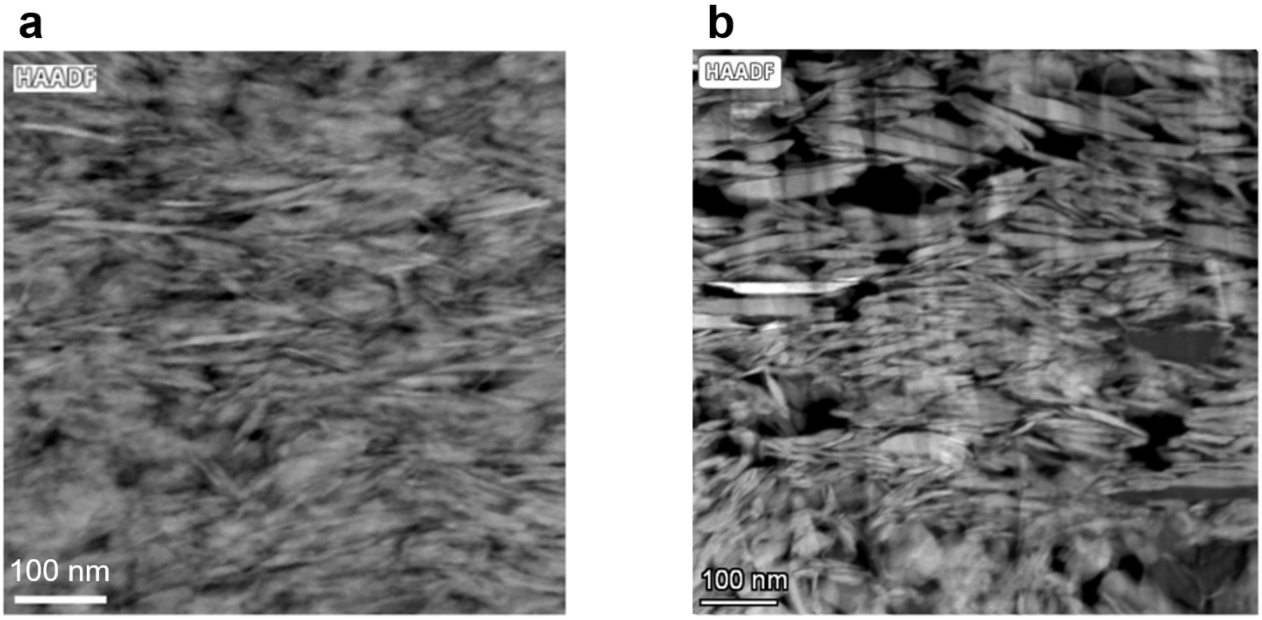

Fig. S12 STEM image of the Ag/MoS_2/Ag memristive switch. (a) unused switch and (b) used switch (20 times on/off cycles). After applying bias on the device, the MoS_2 dielectric layer produces a lot of black blocks, which is a major feature of its topography different from the unused switch.

In Fig. S12, the MoS_2 dielectric layer of the used switches undergoes expansion after the voltage is applied, demonstrating the potential for inducing intrinsic strain within the MoS_2 layer, thereby may lead to a phase change in the MoS_2 lattice structure.

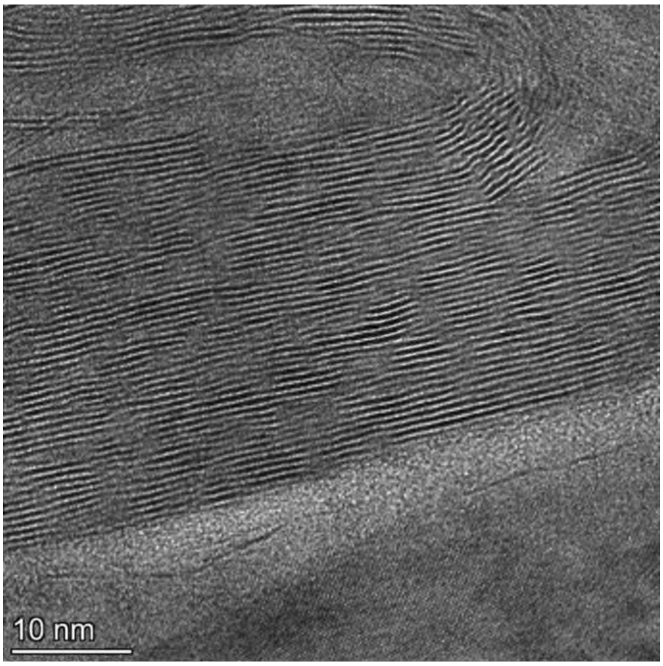

Fig. S13 Bent and strained MoS_2 flake with dislocation paths.

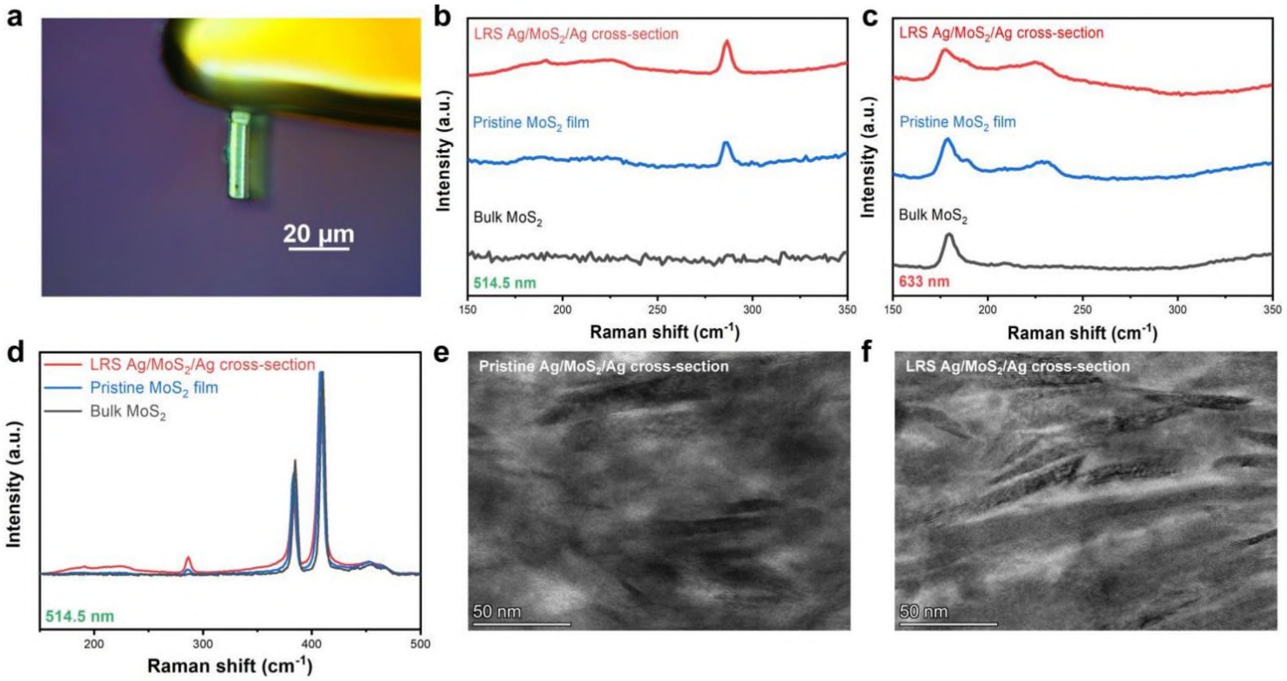

Fig. S14 (a) Optical image of Ag/MoS_2/Ag memristive switch cross section. (b-c) Raman spectra of the MoS_2 cross section taken at 514.5 and 633 nm laser wavelength. (d) Normalized Raman spectra using the peak at 408 cm^{-1}. (e) HRTEM image of the pristine Ag/MoS_2/Ag cross-section. (f) HRTEM image of the Ag/MoS_2/Ag cross-section switched to the LRS.

Raman spectroscopy has been performed on both pristine and used memristive switches on the cross section, by cutting the device with a FIB. The spectrum taken on the switched memristive switch shows a slightly more pronounced peak at 286 cm^{-1}, Fig. S14 (b), which may be attributed to the normally forbidden E1g mode[16.] The relative intensity ratio of the peaks at 226 cm^{-1} and at 406 cm^{-1} is increasing after the memristive switching. This indicates that the MoS_2 flakes are fragmented after biasing, resulting in the reduction in the crystalline region size[17] in agreement with the results presented in Fig. S14 (e), (f) and Fig. S13.

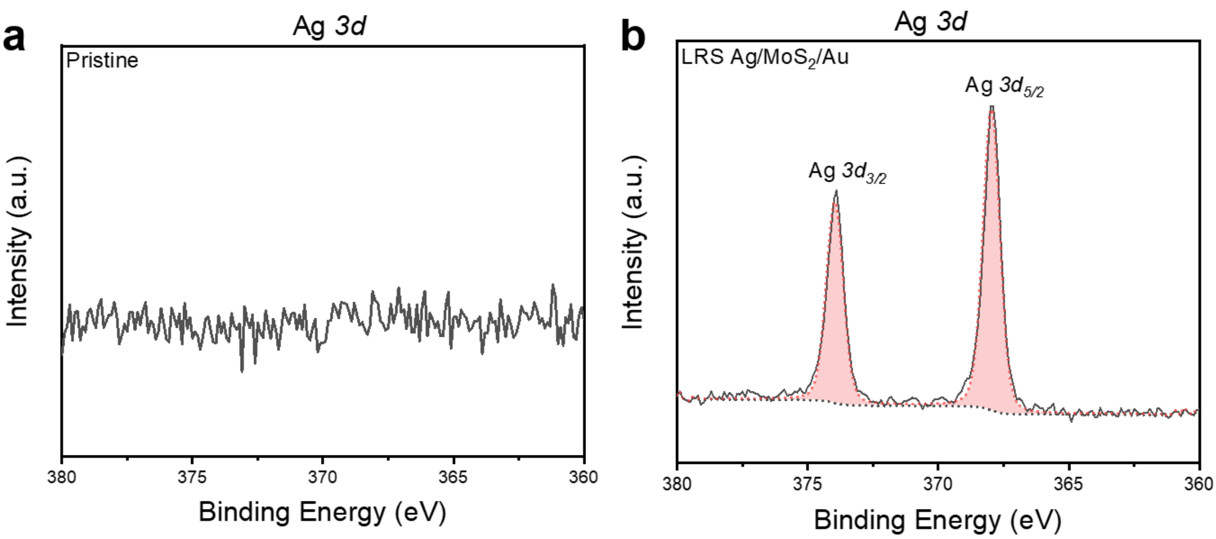

Fig. S15 XPS spectra of Ag peaks of pristine Au/MoS_2/Ag memristive switch (a) and switched Au/MoS_2/Ag memristive switch (b).

To study the Ag migration process through MoS_2 film, we have investigated the changes of Ag *3d* peaks before and after switching the device. A special designed Au/MoS_2/Ag memristive switch which uses Au probe as one of the electrodes was printed. The structure allows us to directly investigate the working interface of MoS_2 after biasing. Fig. S15 shows the XPS spectra for Ag *3d* obtained from both pristine Au/MoS_2/Ag memristive switch and used one (at LRS). As it can be seen, in Fig. S15 (b), prominent Ag *3d* doublets with binding energy values at 367.9 eV is shown, corresponding to Ag^+ $3d_{5/2}$[18]. This indicates that the Ag appeared at the interface of Au/MoS_2, which is clear evidence that the Ag diffuses and migrates through the MoS_2 layer and reaches the top electrode. In addition, Fig. S16 shows the SEM and EDX images of MoS_2/Ag layers after removing the Au probe and biasing. Ag elements can be directly detected from EDX mapping.

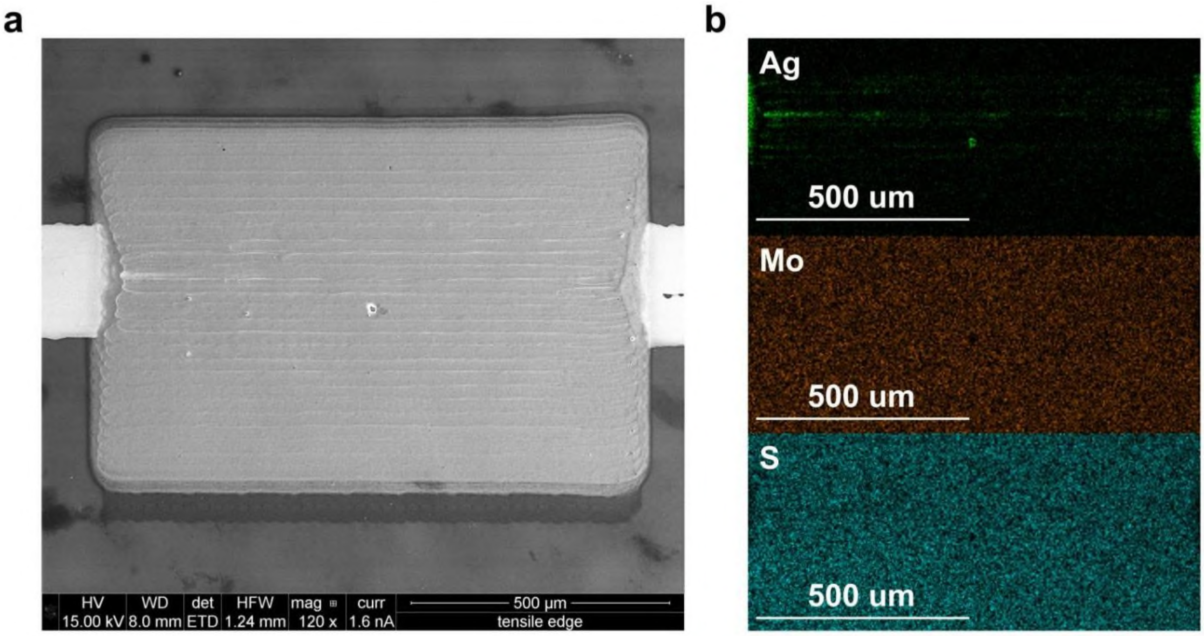

Fig. S16 SEM and EDX image of MoS_2/Ag layers after biasing. (a) SEM image of MoS_2/Ag layers after biasing (b) Ag, Mo and S elements EDX mapping of MoS_2/Ag layers.

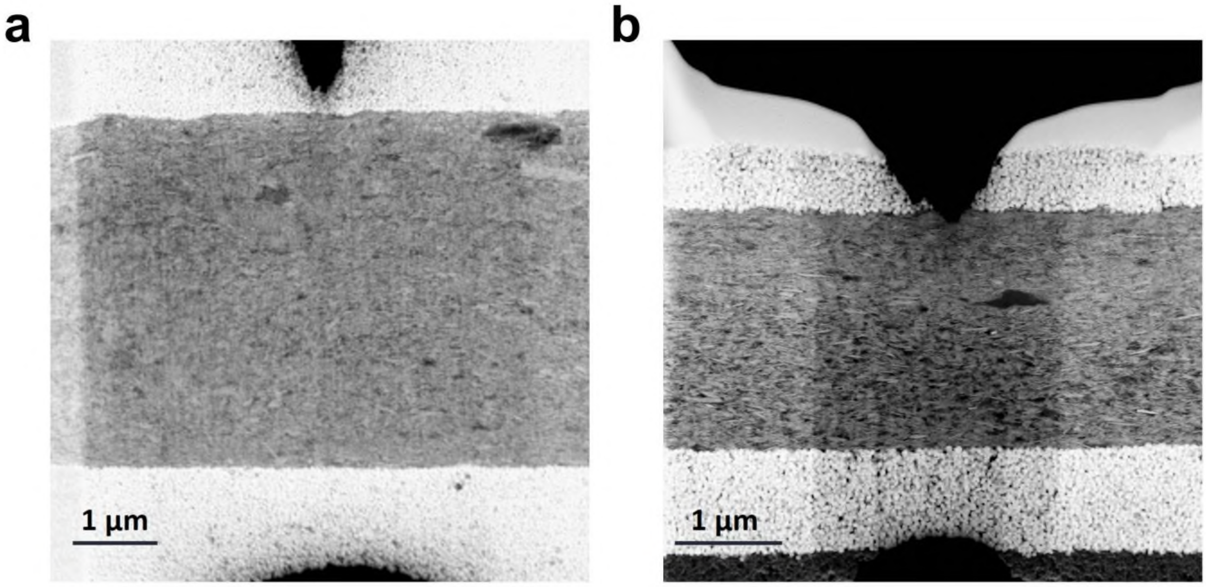

Fig. S17 TEM image of the Ag/MoS_2/Ag memristive switch. (a) unused (pristine) switch and (b) used switch (20 times on/off cycles). After applying bias on the device, the memristive switch shows a less ordered arrangement of MoS_2 flakes in the film and a looser structure, as compared to the pristine film.

S3. Proof-of-concept demonstration of fully printed zero-static power MoS_2 coded RF/microwave reconfigurable graphene metasurface.

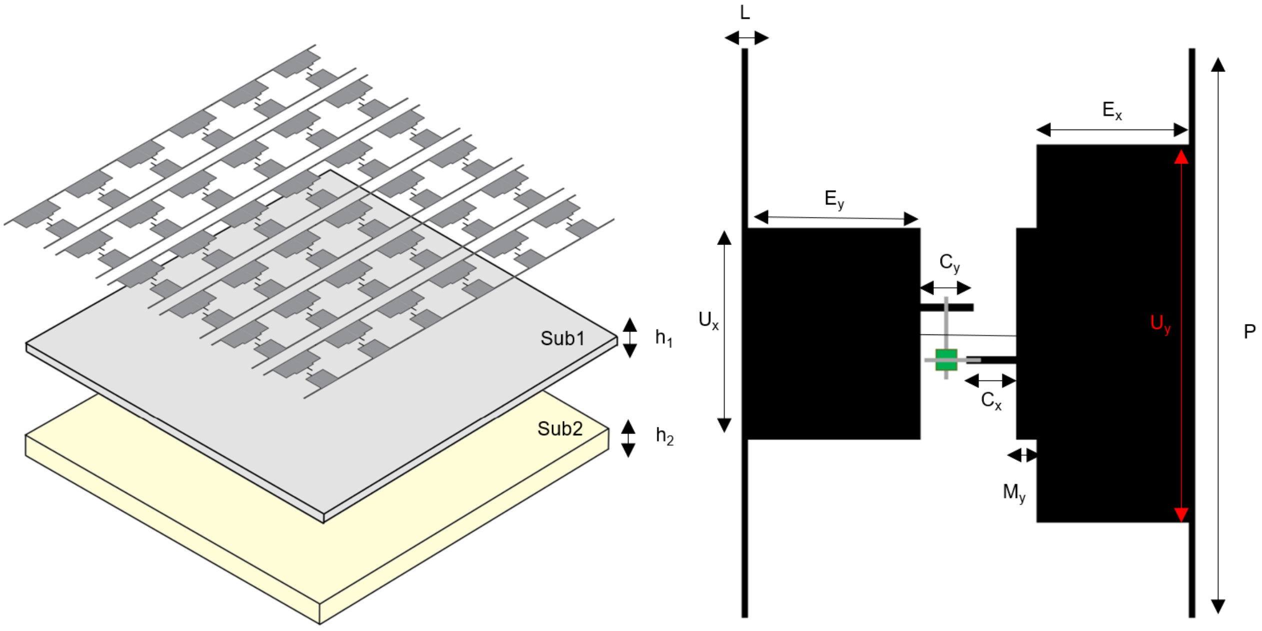

Fig. S18 Dimension of the metasurface.

Table SII. Parameters of the unit cell

parameter	mm	parameter	mm
P	54.4	E_x	14.1
h_1	0.1	E_y	15.9
h_2	6.0	$C_y = C_x$	5.0
m_y	1.8	U_x	18
L	0.2	U_y	36

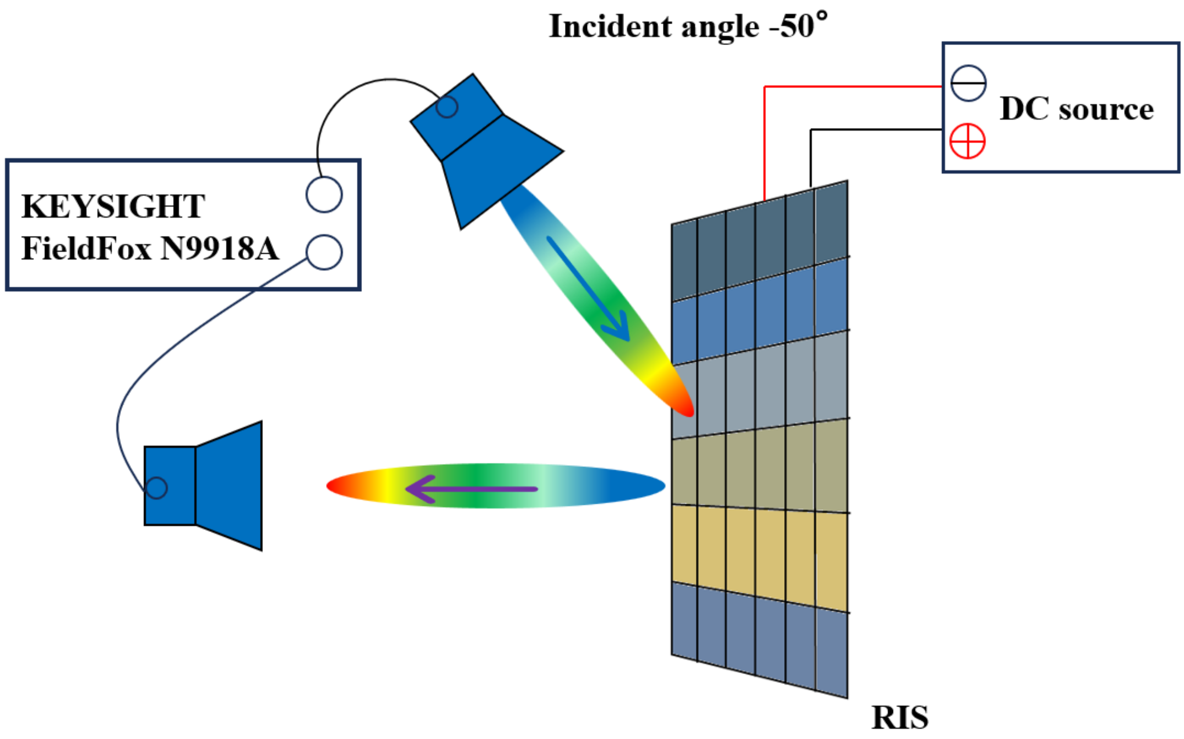

Fig. S19 Illustration of incident wave and how the far field patterns of the metasurface at 3.54 GHz were measured.

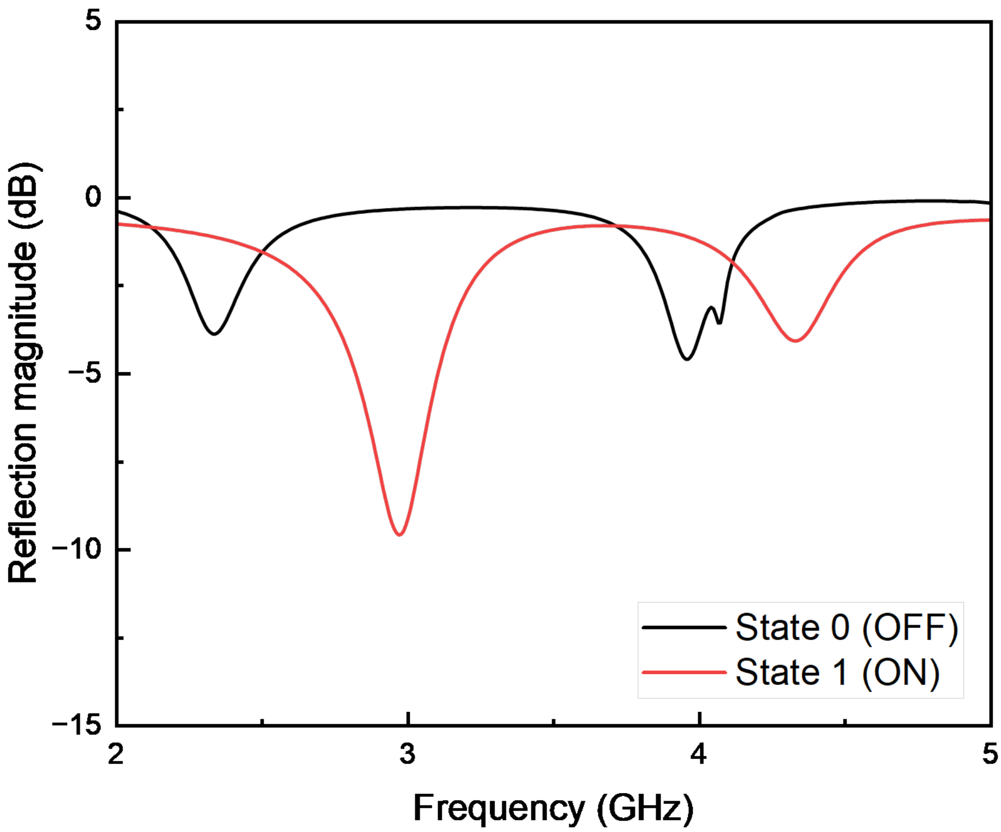

Fig. S20 Reflection magnitude of metasurface unit cell.

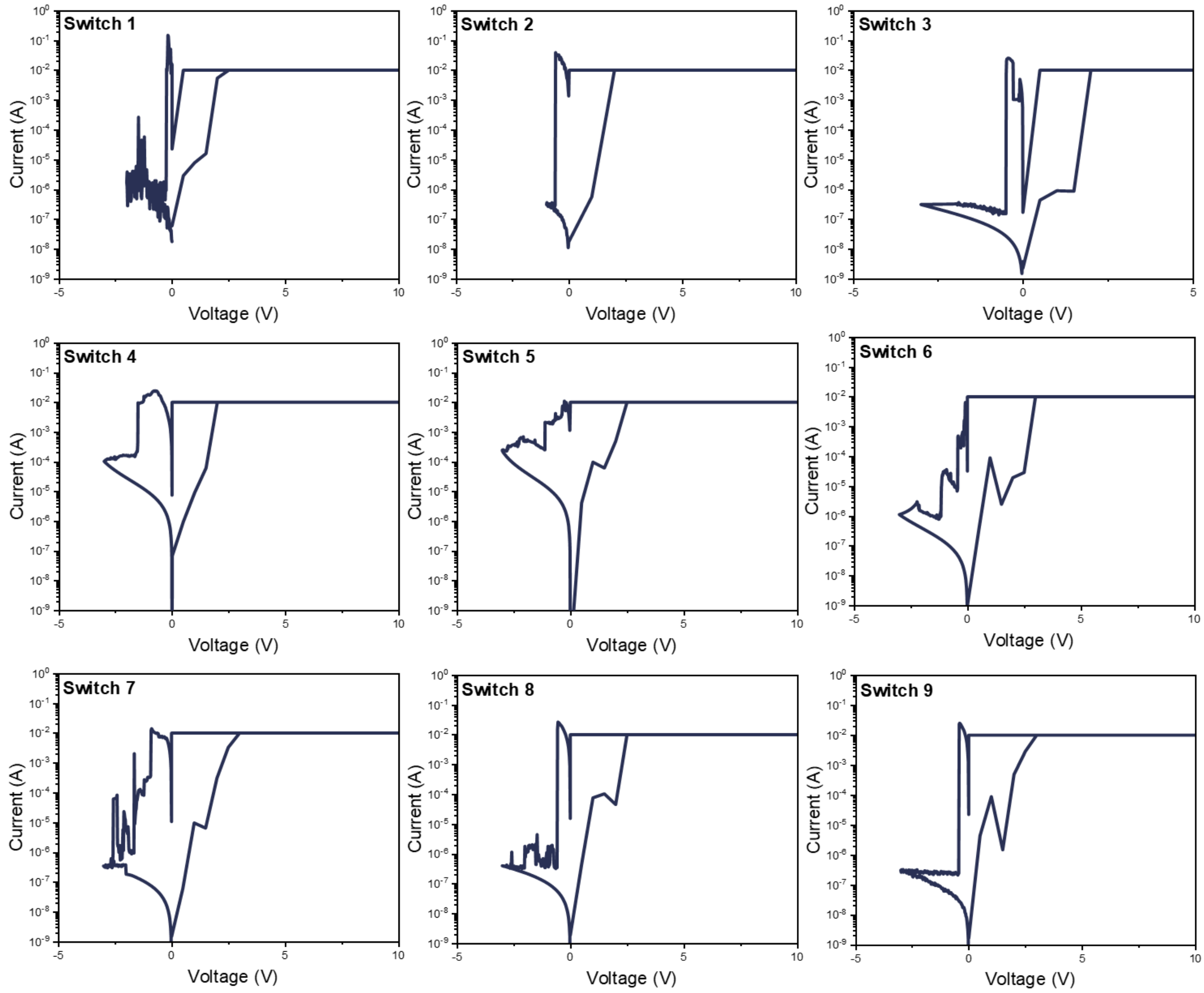

Fig. S21 I-V curve of arrays of the memristive switches used in the reconfigurable metasurface.

Table SIII. Static power consumption comparisons

Ref	Number of Switch	Fabrication process	Static power supply (assuming half working)	Bias	Switch type	Reconfigurability	Material
19	400	PCB technology and layer stacking	5.8 W	5 V	MADP-000907-14020x	Yes	Copper
20	128	PCB technology and layer stacking	0.928 W	1.4 V	MADP-000907-14020W	Yes	Copper
21	160	PCB technology and layer stacking	4 W	0.8V	BAR50-02V	Yes	Copper
22	512	PCB technology and layer stacking	3.7 W	5 V	MADP-000907-14020x	Yes	Copper
23	18	PCB technology and layer stacking	1.08 W	5 V	BAR63-03W)	Yes	Copper
24	1152	PCB technology and layer stacking	69.12 W	5 V	MADP-000907-14020x	Yes	Copper
Our work	9	Fully printed	0 W	1.7 V	Non-volatile MoS_2 switch	Yes	Graphene

References

1. Choi, K. H., Mustafa, M., Rahman, K., Jeong, B. K. & Doh, Y. H. Cost-effective fabrication of memristive devices with ZnO thin film using printed electronics technologies. *Appl. Phys. A* 106, 165–170 (2012).

2. Muhammad, N. M. *et al.* Fabrication of printed memory device having zinc-oxide active nano-layer and investigation of resistive switching. *Curr. Appl. Phys.* 13, 90–96 (2013).

3. Duraisamy, N., Muhammad, N. M., Kim, H.-C., Jo, J.-D. & Choi, K.-H. Fabrication of TiO_2 thin film memristor device using electrohydrodynamic inkjet printing. *Thin Solid Films* 520, 5070–5074 (2012).

4. Siddiqui, G., Ali, J., Doh, Y.-H. & Choi, K. H. Fabrication of zinc stannate based all-printed resistive switching device. *Mater. Lett.* 166, 311–316 (2016).

5. Catenacci, M. J. *et al.* Fully printed memristors from Cu–SiO_2 core–shell nanowire composites. *J. Electron. Mater.* 46, 4596–4603 (2017).

6. Salonikidou, B. *et al.* Inkjet-printed Ag/a-TiO_2/Ag neuromorphic nanodevice based on functionalized ink. *Adv. Eng. Mater.* 24, 2200439 (2022).

7. Rafique, A. F., Haji Zaini, J., Bin Esa, M. Z. & Nauman, M. M. Printed memory devices using electrohydrodynamic deposition technique. *Appl. Phys. A* 126, 134 (2020).

8. Nauman, M. M., Zulfikre Esa, M., Zaini, J. H., Iqbal, A. & Bakar, S. A. Zirconium oxide based memristors fabrication via electrohydrodynamic printing. In *2020 IEEE 11th International Conference on Mechanical and Intelligent Manufacturing Technologies (ICMIMT)* 167–171 (IEEE, 2020).

9. Zhu, K. *et al.* Inkjet-printed h-BN memristors for hardware security. *Nanoscale* 15, 9985–9992 (2023).

10. Feng, X. *et al.* A fully printed flexible MoS_2 memristive artificial synapse with femtojoule switching energy. *Adv. Electron. Mater.* 5, 1900740 (2019).

11. Li, Y. et al. Aerosol jet printed WSe_2 crossbar architecture device on kapton with dual functionality as resistive memory and photosensor for flexible system integration. *IEEE Sens. J.* 20, 4653-4659 (2020)

12. Lien, D.-H. *et al.* All-printed paper memory. *ACS Nano* 8, 7613–7619 (2014).

13. Awais, M. N., Kim, H. C., Doh, Y. H. & Choi, K. H. ZrO_2 flexible printed resistive (memristive) switch through electrohydrodynamic printing process. *Thin Solid Films* 536, 308-312 (2013)

14. Khan, M. *et al.* All-printed flexible memristor with metal–non-metal-doped TiO_2 nanoparticle thin films. *Nanomaterials* 12, 2289 (2022).

15. Peng, Z. et al. Fully printed memristors made with MoS_2 and graphene water-based inks. *Mater. Horiz.* 11, 1344-1353.(2024).

16. Frey, G. L. et al. Raman and resonance Raman investigation of MoS_2 nanoparticles. *Phys. Rev. B* 60, 2883 (1999).

17. Blanco, É. et al. Resonance Raman spectroscopy as a probe of the crystallite size of MoS_2 nanoparticles. *CR. Chim.* 19, 1310-1314 (2016).

18. Gaarenstroom, S. W. & Winograd N. Initial and final state effects in the ESCA spectra of cadmium and silver oxides. *J. Chem. Phys.* 67, 3500-3506 (1977).

19. Gros, J.-B., Popov, V., Odit, M. A., Lenets, V. & Lerosey, G. A reconfigurable intelligent surface at mmWave based on a binary phase tunable metasurface. *IEEE Open J. Commun. Soc.* 2, 1055–1064 (2021).

20. Wang, Z. X. *et al.* A low-cost and low-profile electronically programmable bit array antenna for two-dimensional wide-angle beam steering. *IEEE Trans. Antennas Propag.* 71, 342–352 (2023).

21. Trichopoulos, G. C. *et al.* Design and evaluation of reconfigurable intelligent surfaces in real-world environment. *IEEE open j. Commun. Soc.* 3, 462-474 (2022).

22. Bai, X. *et al.* Radiation-type programmable metasurface for direct manipulation of electromagnetic emission. *Laser Photonics Rev.* 16, 2200140 (2022).

23. Chaimool, S., Hongnara, T., Rakluea, C., Akkaraekthalin, P. & Zhao, Y. Design of a PIN diode-based reconfigurable metasurface antenna for beam switching applications. *Int. J. Antennas Propag.* 2019, 7216324 (2019).

24.Wang, C. et al. Reconfigurable transmissive metasurface synergizing dynamic and geometric phase for versatile polarization and wavefront manipulations. *Mater. Design.* 225, 111445 (2023).
