# Supplementary material for: Fully printed zero-static power MoS2 switch coded reconfigurable graphene metasurface for RF/microwave electromagnetic wave manipulation and control
Source: Nat Commun. 2024 Dec 4;15:10591. doi: 10.1038/s41467-024-54900-z (PMC11618370; doi:10.1038/s41467-024-54900-z)
Supplement: Supplementary file 3 — Description of Additional Supplementary Files [file 41467_2024_54900_MOESM3_ESM.pdf]

Description of Additional Supplementary Files

File Name: Supplementary Data 1

Description: This file includes the experimental data discussed in our main text, encompassing DC and RF properties, material characterization, and the far-field pattern of the metasurface.
